# Patterns of Scientific and Clinical Impact in Cancer Randomized Clinical Trials

**DOI:** 10.1001/jamanetworkopen.2022.19657

**Published:** 2022-06-30

**Authors:** Van T. Nghiem, Riha Vaidya, Joseph M. Unger

**Affiliations:** 1 University of Alabama at Birmingham School of Public Health, Birmingham; 2 Fred Hutchinson Cancer Research Center, SWOG Statistics and Data Management Center, Seattle, Washington

## Abstract

This cohort study examines whether randomized clinical trials of cancer treatment with negative findings that inform cancer care guidelines are recognized for their scientific relevance by the research community.

## Introduction

Findings from randomized clinical trials of cancer treatments represent the highest level of evidence for informing cancer care guidelines. Trials with both positive and negative findings can influence guideline care recommendations.^1^ This highlights the importance of completing all trials successfully and demonstrates how trials with negative findings are critical for understanding which newly developed treatments should not be used.^2^ One question is whether negative trials that influence guideline care are recognized for their scientific relevance by the research community.

## Methods

The requirement for institutional review board review and informed consent was waived because only data from published clinical trials that previously obtained institutional review board approval and informed consent were used, in accordance with 45 CFR §46. This cohort study follows the Strengthening the Reporting of Observational Studies in Epidemiology (STROBE) reporting guideline for cohort studies.

We examined how often positive and negative trials informed guideline care recommendations and their scientific impact. We evaluated phase 3 randomized clinical trials of cancer treatment from the SWOG Cancer Research Network activated from 1980 onward. A practice-influential (PI) trial was defined as one whose findings supported recommended treatment in National Comprehensive Cancer Network clinical guidelines or were cited in package inserts for new drug approvals from the US Food and Drug Administration.^1^ Determinations were made by consensus among all authors. Positive trials were those for which the group receiving the experimental regimen had statistically significantly better results than the standard treatment group according to the protocol-specified primary end point; trials with null or negative findings were deemed negative.^3^ Scientific impact was determined by annual citation counts through 2021 from Google Scholar for the primary trial report.^4^ Included trials were published 8 years or more before this analysis to allow assessment of clinical and scientific impact.

Generalized estimating equations for Poisson regression were used to model annual citation counts for up to 10 years after publication.^5^ Relative risk (RR) estimates (ie, the ratio of annual citation rates) and 95% CIs were determined. Model variables included postpublication time, PI vs non-PI status, positive vs negative trial outcome, and publication before vs after 2001 (median publication year). Data analysis was performed from February to May 2022. We used SAS statistical software version 9.4 (SAS Institute) with a 2-sided significance level of α = .05.

## Results

Overall, 164 RCTs published between 1986 and 2013 with 116 449 patients were included. The most common cancers were breast (25 trials [15.2%]) and lung (23 trials [14.0%]) (Table). One-third (54 trials [32.9%]) were adjuvant trials; 160 trials (97.6%) included intervention with systemic therapy.

**Table.** Characteristics of Phase 3 Cancer Clinical Trials in the SWOG Cancer Research Network

Characteristics	All studies, No. (%) (N = 164)
Cancer type	
Breast	25 (15.2)
Gastrointestinal	20 (12.2)
Genitourinary	22 (13.4)
Gynecological	11 (6.7)
Head and neck	7 (4.3)
Leukemia	16 (9.8)
Lung	23 (14.0)
Lymphoma	12 (7.3)
Melanoma	9 (5.5)
Myeloma	7 (4.3)
Others^a^	12 (7.3)
Study setting	
Adjuvant	54 (32.9)
Advanced	110 (67.1)
Primary end point type	
Overall survival	39 (23.8)
Multiple (including overall survival)	116 (70.7)
Others	9 (5)
Treatment groups, No.	
2	107 (65.2)
>2	57 (34.8)
Intervention included	
Systemic therapy	160 (97.6)
Biologic therapy	20 (12.2)
Surgery	18 (11.0)
Radiation therapy	41 (25.0)
Transplant	12 (7.3)
Blinded treatment	4 (2.4)
Study design	
Superiority	158 (96.3)
Noninferiority or equivalence	6 (4)
Intergroup study	
Yes	115 (70.1)
No	49 (29.9)
Lead group	
SWOG led	87 (53.0)
Other group led	77 (47.0)
Final accrual for all studies, mean (SD), No. of participants	710.0 (1009.3)
Time from activation to publication for all studies, mean (SD), y	9.4 (3.4)
Time from completion to publication for all studies, mean (SD), y	5.0 (2.6)
Year of trial completion	
1980-1989	29 (17.7)
1990-1999	84 (51.2)
2000 or after	51 (31.1)

^a^ Includes sarcoma, brain cancer, germ cell tumor, and myelodysplastic syndrome.

Seventy-six five trials (46.3%) influenced care guidelines; 64 supported National Comprehensive Cancer Network guidelines, 6 supported Food and Drug Administration new drug approvals, and 6 supported both. The observed mean (SD) annual citation rate was 65.5 (67.8) for PI trials and 16.2 (17.7) for non-PI trials. The generalized estimating equation model identified a quadratic trend over time (Figure). The model-estimated RR for PI vs non-PI trials was 3.0 (95% CI, 2.2-4.2; *P* < .001).

**Figure.**
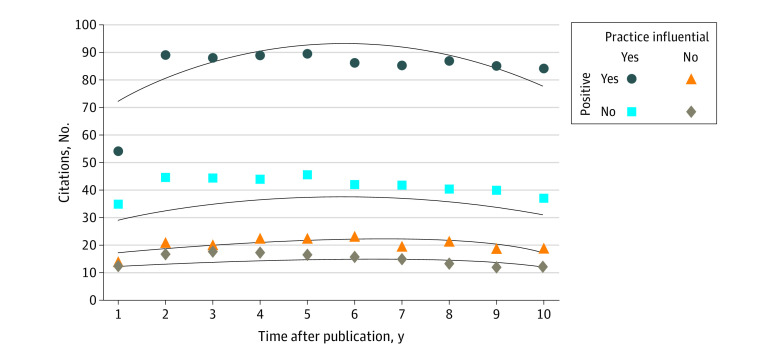
Estimated Mean Annual Citation Count (Lines) vs Observed Mean Annual Citation Count (Points) by Year After Publication by Trial Subgroup

Sixty trials (36.6%) were positive and 104 (63.4%) were negative. The observed mean (SD) annual citation rate was 66.0 (76.0) for positive trials and 23.5 (24.6) for negative trials (model-adjusted RR, 2.2; 95% CI, 1.6-3.1; *P* < .001). Considering both factors, the highest mean (SD) annual citation counts were among positive PI trials (82.7 [82.0] citations; 44 studies [26.8%]), then negative PI trials (41.8 [28.3] citations; 32 studies [19.5%]), then positive, non-PI trials (20.2 [18.6] citations; 16 studies [9.8%]), and finally negative non-PI trials (15.4 [17.5] citations; 72 studies [43.9%]) (Figure). Negative PI trials had 1.7 times (95% CI, 1.0-2.9; *P* = .047) greater RR of annual citation than positive non-PI trials.

## Discussion

The findings of this cohort study reaffirm that positive trials are more commonly cited than negative trials^3^ and newly demonstrate that PI trials are cited much more than non-PI trials. Importantly, negative PI trials were cited more than positive non-PI trials. How common a cancer is may be associated with citation patterns, representing a potential study limitation. Nonetheless, the findings highlight the value of negative trials to clinical research and the critical need to publish all clinical research studies, regardless of their outcome, given their importance in advancing the understanding of cancer treatments and their influence on clinical care recommendations, which guide patient care.

## References

[1] Unger JM, Nghiem VT, Hershman DL, Vaidya R, LeBlanc M, Blanke CD. Association of National Cancer Institute–sponsored Clinical Trial Network group studies with guideline care and new drug indications. JAMA Netw Open. 2019;2(9):e1910593. doi:10.1001/jamanetworkopen.2019.10593 31483471 PMC6727679

[2] Huntington SF, Gross CP. Negative studies in cancer research: why the negativity? JAMA Oncol. 2016;2(7):865-866. doi:10.1001/jamaoncol.2015.6540 26967116

[3] Unger JM, Barlow WE, Ramsey SD, LeBlanc M, Blanke CD, Hershman DL. The scientific impact of positive and negative phase 3 cancer clinical trials. JAMA Oncol. 2016;2(7):875-881. doi:10.1001/jamaoncol.2015.6487 26967260 PMC4945370

[4] Narin F. Evaluative Bibliometrics: The Use of Publication and Citation Analysis in the Evaluation of Scientific Activity. National Science Foundation; 1976.

[5] Liang KY, Zeger SL. Longitudinal data analysis using generalized linear models. Biometrika. 1986;73(1):13-22. doi:10.1093/biomet/73.1.13

